# Points, skyrmions and torons in chiral nematic droplets

**DOI:** 10.1038/srep26361

**Published:** 2016-05-20

**Authors:** Gregor Posnjak, Simon Čopar, Igor Muševič

**Affiliations:** 1 J. Stefan Institute, Condensed matter department, Ljubljana, Slovenia; 2 Faculty of mathematics and physics, University of Ljubljana, Ljubljana, Slovenia

## Abstract

Chiral nematic droplets with perpendicular surface alignment of liquid crystalline molecules frustrate the helical structure into convoluted 3D textures with complex topology. We observe the droplets with fluorescent confocal polarising microscopy (FCPM), and reconstruct and analyse for the first time the topology of the 3D director field using a novel method of director reconstruction from raw data. We always find an odd number of topological defects, which preserve the total topological charge of the droplet of +1 regardless of chirality. At higher chirality, we observe up to 5 point hedgehog defects, which are elastically stabilized with convoluted twisted structures, reminiscent of 2D skyrmions and toron-like structure, nested into a sphere.

Topological properties of nematic liquid crystal colloids have been in the research focus during the last decade because of their rich topology and ease of control, manipulation and observation in real space and time. Based on topological defects, new liquid crystal materials have been developed, where the topological conservation laws protect their structure, such as 2D and 3D nematic colloidal crystals^1^,^2^, entangled^3^,^4^, linked and knotted colloidal superstructures^5^,^6^. These new materials combine topology, fluidity, softness, and high response to external fields, which gives rise to their extraordinary material properties.

Nematic and chiral nematic droplets with well defined surface orientation of LC molecules are one of the earliest realizations of a topologically rich LC system. When LC is confined into a sphere, the topological constraints require formation of topological defects, which were observed under an optical microscope as surface hedgehogs for parallel surface anchoring of non-chiral NLC molecules^7^,^8^,^9^ and point monopoles or closed defect rings for perpendicular surface anchoring^10^,^11^,^12^. Topological complexity becomes even richer in topologically non-trivial space, such as in the NLC confined to handle-bodies, as observed recently^13^. On the other hand, the chirality of a nematic liquid crystal is also known to enhance the richness of topological phenomena and this was studied in a number of analytical^12^,^14^,^15^ and numerical studies^16^. In chiral nematic droplets with perpendicular surface anchoring, knotted topological defects have been predicted by numerical simulations with fully tensorial Landau-de Gennes approach^17^.

Chiral nematic droplets with parallel surface anchoring were intensively studied in the experiments^10^,^11^,^12^,^18^,^19^,^20^, because this surface anchoring causes little structural frustration and the spatially periodic chiral nematic structure can easily be nested into the sphere forming an onion-like periodic structure. The helical order evolves smoothly from the centre to the surface with a twisted or double-twisted topological line defect penetrating the droplet half-way or through the entire droplet. In contrast to theory, there are only several experimental studies of topological defects in chiral nematic droplets with perpendicular surface anchoring^10^,^19^,^21^, because this boundary condition induces strong frustration of the director field. Some of the predicted complexity of chiral nematic droplets with perpendicular surface anchoring was reported in a recent optical polarization microscopy study^21^. However, there was no attempt to reconstruct the director field inside chiral nematic droplets from the optical polarization images solely and the question of topology remained open. As concluded by the authors^21^, care has to be taken in interpreting simple optical polarization images because of well known optical artefacts arising from polarization and lensing effects in birefringent inhomogeneous media such as chiral nematic droplets. An exact experimental method is therefore needed to measure and reliably reconstruct the structure and topology of chiral nematic droplets.

Fluorescent confocal polarizing microscopy (FCPM) is a powerful optical technique, which is used to determine the orientation of fluorescent dye molecules in liquid crystals^22^,^23^ and in principle allows for the reconstruction of a 3D director field. FCPM was used to image cholesteric defect structures in Cano-wedges^24^, and director fields in topologically complex objects, like the Hopf fibration and torons^25^,^26^,^27^, around colloidal handlebodies^28^ and microposts^29^. In all cases, the director was reconstructed indirectly from the measured FCPM intensities either by hand^24^, by visually comparing the measured and simulated FCPM intensities^25^ or by observing the orientation of the in-plane director component on a selected intensity iso-surface^26^. These indirect approaches therefore do not take measured FCPM intensities as an input for a numerical algorithm, which would automatically and independently of any external action generate a genuine reconstructed 3D director profile. They can work well with relatively simple systems with some level of symmetry and known orientation of the structure under investigation. However, in more complicated structures with complex topological defects in 3D, it is not possible to deduce the director manually. Chiral nematic droplets are exactly this case, and recent studies^17^ clearly stressed the need for direct and reliable reconstruction of director fields from experimental data.

Here we present for the first time full reconstruction of 3D director fields and topological defects from FCPM imaging using a numerical approach for director reconstruction from fluorescence 3D images at different polarizations. The reconstructed 3D director fields inside chiral nematic droplets with variable chirality allow for the full analysis of the structure of defects, their topological characterisation and the analysis of arbitrary cross sections of reconstructed 3D director fields. For low chirality, we observe an isolated point hedgehog defect, which gets accompanied with additional defect-antidefect pairs at increasing chirality. We always find an odd number of defects, which preserves the total topological charge of the droplet of +1 in all cases and topologies. We observe up to 5 point hedgehog defects, which are elastically stabilized in a droplet with convoluted twisted structures, reminiscent in their cross sections to 2D skyrmions and in some cases forming a toron-like structure, nested into a sphere.

## Experiment

### Fluorescent confocal polarising microscopy

FCPM^22^ uses a confocal microscope^30^ to achieve 3D resolution by collecting fluorescent light from anisotropically shaped dye molecules, which are added to the LC. In some cases, the fluorescence can be collected from the LC molecules themselves using nonlinear optical microscopy and multiphoton excitation to induce fluorescence^31^,^32^. In standard FCPM, rod-like dye molecules are added to a LC phase of rod-like molecules, and the long axes and the emission dipoles of dye molecules are usually aligned with the LC director^33^. When linearly polarised light is used for excitation of the dye and a polariser with the same polarisation for detection of fluorescence, the detected fluorescent intensity depends on the angle between the polarisation and the director field which gives rise to a contrast in fluorescence intensity. By measuring the fluorescence intensity at several linear polarisations of excitation and detection, it is possible to determine the orientation of local director in a plane perpendicular to the optical axis (the angle ***ϕ*** defining the orientation of dye molecules within the imaging, i. e. *xy* plane^26^) and also the size of the component parallel to the optical axis (the *z* component, i. e. the out-of-imaging plane component). The relation between the sum of measured intensities of the fluorescent signal at different polarisations *I*_exp_ and the angle ***θ*** between the director and the *xy* plane is highly non-linear:


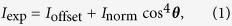


where *I*_offset_ is a background due to sample imperfections and measurement bias and *I*_norm_ is the normalisation of fluorescence intensity.

Although FCPM and its derivatives are superior and unique methods that allow for reconstruction of director field in liquid crystals, their application requires care to avoid well known optical artefacts arising from aberrations and lensing due to curved interfaces and refractive index mismatch, dye accumulation at interfaces and in defect cores, polarization guiding and other birefringence effects^34^,^35^. For this reason LCs with low birefringence are used, doped with dyes with good alignment in LCs in very low concentrations, and the interfaces between the LC and bounding media have to be closely index matched (in our case the refractive index mismatch is less than 0.01). Even with experimental optimization, the most serious problem of FCPM is an inherent ambiguity in director reconstruction. Because the polarisation of the FCPM scanning beam always lays in a plane perpendicular to the optical axis, FCPM cannot distinguish between positive and negative vertical components of the director. The director field can therefore be only partially determined as **n** = (cos ***θ*** cos ***ϕ***, cos ***θ*** sin ***ϕ***, ±sin ***θ***). One can in principle distinguish between the two ambiguous orientations by tilting the sample^36^ but the contrast depends strongly on the angle between the imaging polarisation and the director. This method works for simple sample topology and confinement, but is highly impractical because of experiment duration and complexity of reconstruction when imaging LC with complex topology, such as chiral nematic droplets with knotted and linked director fields, which we are targeting.

### Determining the sign of *n_z_* by simulated annealing

Because single sample tilt FCPM cannot determine the correct sign of the z-component of the director field, we are left with two possibilities for each measuring voxel in the sample: (*n*_*x*_, *n*_*y*_, *n*_*z*_) and (*n*_*x*_, *n*_*y*_, −*n*_*z*_). This gives 2^*M*^ possible configurations, where *M* is the number of sampled voxels in the droplet. Because the elastic energy of the LC droplet should be minimal, the problem is analogous to finding a stable configuration of a two-level system, such as a system of $M$ 1/2 spins, interacting with each other. If we consider the voxel in the LC droplet, from which the fluorescent intensity is being collected, the director is locally tilted with respect to the optical axis of the microscope by ***θ***. However, there are two possible tilts, i.e. +***θ*** and −***θ***, both of which give the same fluorescence intensity. This corresponds to two possible orientations of the director with *n*_*z*_ component either up or down. The elastic energy of interaction of these two states with neighbors will be different, which makes our system of $M$ interacting voxels in a droplet formally equivalent to the $M$ interacting 1/2 spins. Minimization of the free energy of such a system is therefore an optimisation problem, which can be solved by using a simulated annealing algorithm. It was first used for optimising the placement and wiring of components in electronic systems^37^.

We implemented an algorithm that decides to change or not to change the sign of *n*_*z*_ of a randomly selected voxel in the droplet (or any other structure) depending on the difference in elastic energy of the “up” and “down” states. The elastic energy is calculated from


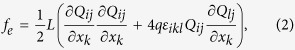


where *L* is the elastic constant in the one constant approximation and *q* = 2*π*/*p*_0_ is the inverse cholesteric pitch *p*_0_, which makes the elastic energy chirality-dependent. The tensor *Q* is calculated from the director as


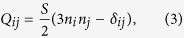


where the scalar order parameter *S* is taken to be constant. Equation (2) is discretised on a cubic grid, each point of which corresponds to a voxel of experimental data and the derivatives are approximated with a mixture of symmetrical and non-symmetrical finite differences, which increases the numerical stability of the algorithm. If the elastic energy is decreased in the flipped state, the algorithm accepts the change, otherwise the change is accepted with a probability given by the Boltzmann weight exp(−Δ*E*/*t*), where Δ*E* is the difference in the elastic energy caused by the flip, and *t* is a free parameter which acts as an effective temperature. The acceptance of unfavourable configurations with a probability depending on a free parameter helps the algorithm not to become stuck in local minima and thus to scan the configuration space much more efficiently. The algorithm is left to run until the energy of the whole configuration at a given temperature is stabilised before reducing the temperature. The annealing process starts at a high *t* randomising the signs of the $z$ components and is then slowly lowered allowing the structure to find a configuration with low energy at each step. A fixed director field with a radial configuration is added outside the droplet, which acts as an effective perpendicular molecular alignment layer in the surface voxels of the droplet through the derivative terms in equation (2). The annealing algorithm stops when energy stabilises at the last temperature in the annealing schedule. Typical calculation times of our algorithm for *M* ~ 10^6^ sample points (droplets with 10–15 μm diameter and sampling of 120 nm) were 5 minutes on an Intel i7 desktop computer with no parallelisation.

The algorithm was first tested on ideal chiral nematic structures in droplets, which were obtained by numerical minimization of the Landau-de Gennes free energy on a finite grid and exhibited ideal optical contrast (t. i. areas with vertical director had 0 intensity and with horizontal maximum intensity). The algorithm worked nearly deterministically on such director fields, failing only when the *n*_*z*_ was very close to 0 or 1 – in other words, where the energy difference between the + and − state was negligible and therefore there were no artefact domain walls in the annealed structures. In unfavourable experimental cases some domains could potentially become trapped during the annealing and domain walls would remain even after the thermalisation. The numerically simulated chiral droplets were also compared directly with the annealed experimental data, obtained from droplets with identical topology, and good qualitative matching of the topology and geometry of complex structures was observed.

### Algorithm calibration on simple topology

**Figure 1:**
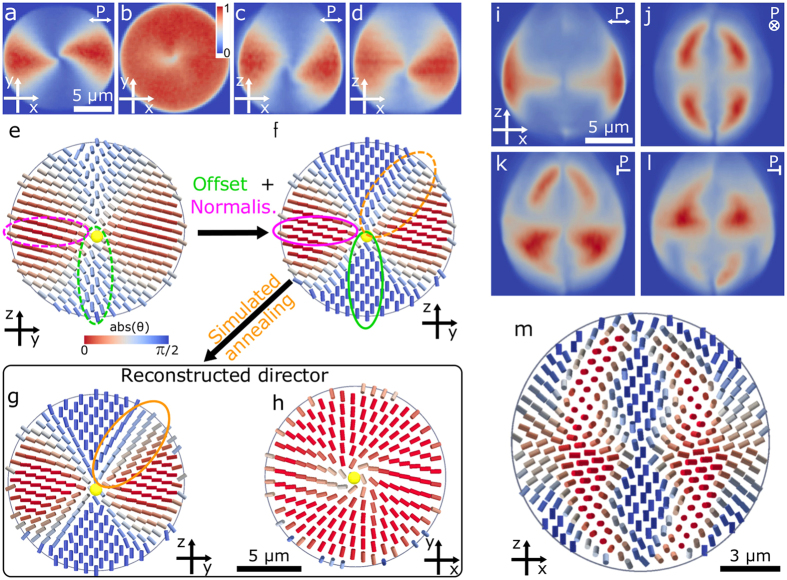
Director reconstruction from FCPM images of a radial nematic droplet. (**a**–**d**) Experimental FCPM intensities in different planes. In (**a**,**c**), the FCPM intensity of a single polarisation P is shown and in (**b**,**d**) the total FCPM intensity *I*_exp_. (**e**–**g**) The director field, reconstructed from data in (**a**–**d**). (**e**) The director is reconstructed without FCPM intensity normalisation, no offset correction and no z-component sign correction. (**f**) The FCPM intensity is normalised and offset is corrected. (**g**) All corrections are applied. (**h**) The reconstructed director field in the xy plane going through the point defect (yellow dot). (**i**–**l**) FCPM intensities taken at 4 different excitation/emission polarisations in the same plane in a complex cholesteric droplet. (**m**) Numerically reconstructed director field from experimental data in (**i**–**l**). Cylinders in (**e**) are coloured by *I*_exp_ and in (**f**–**h**,**m**) by *I*_norm_ i.e. the size of the director projection on the *xy* plane.

First we reconstructed the director inside a radial, non-chiral nematic droplet to calibrate and test the algorithm on real experimental data. Raw FCPM 3D images of droplets were taken at four different polarizations, and were corrected for dye bleaching, aberrations, absorption of the excitation light, and background light intensity. Figure 1a–d show experimental intensities after bleaching correction and aberration correction by deconvolution. The intensities taken at a single polarisation (Fig. 1a,c) show radial direction of the projection of director field on the *xy* plane. The total intensity *I*_exp_ (Fig. 1b,d), which is proportional to the projection of director field on the *xy* plane, shows that the director is in-plane around the equator, which agrees with the expected radial director configuration.

A background light intensity *I*_offset_ was subtracted from the sum of raw intensities *I*_exp_ and the data were normalised with *I*_norm_. The background *I*_offset_ is caused by imperfect ordering of the dye, high numerical aperture of the microscope objective, scattering in the LC and other imperfections. The ratio between maximum and minimum *I*_exp_ is between 2 and 3. Therefore if a background is not subtracted, *I*_offset_ causes areas of vertical director to be calculated as director at an oblique angle, as seen in the area marked with a green ellipse in Fig. 1e. In such areas the angle ***ϕ*** is ill-defined and if ***θ*** is not corrected to vertical, the director is noisy, which hinders the success of the annealing algorithm. The reconstruction process is therefore more stable, if the offset in dark areas is taken about 10% higher that the actual level of the fluorescent intensity in those parts. After subtracting the offset from the signal, the obtained values of intensity are negative in some dark areas and there the intensity is set to 0.

A normalization of the fluorescent signal is needed because absorption, scattering and optical effects, such as refraction and spherical aberration, strongly attenuate the detected fluorescence intensity, which causes the maximum *I*_exp_ in *xy*-layers to decrease significantly with increasing *z* coordinate. Without correction, the reconstructed director tilts away from the imaging plane, as seen in the area marked with a magenta ellipse in Fig. 1e. In such areas the transition between negative and positive correctly oriented ***θ*** would result in finite director jumps, which would obstruct the annealing algorithm. Because of this it is favourable to set the normalization value about 5% below the actual fluorescence intensity in the brightest regions and to set corrected intensities which are larger than 1, to 1. By doing so, the experimental data is clipped to the 0–1 intensity range.

Both *I*_norm_ and *I*_offset_ vary with the depth of scanning *z* and have to be determined from experimental data. After applying the corrections ***θ*** is calculated. The clipping of the intensity causes artefacts which appear as areas of completely horizontal or vertical director (marked by a magenta and a green ellipse in Fig. 1f), but as the transients between these regions stay where they should be, the topology of the director field is not affected. The background and normalisation corrections affect only the out of plane angle ***θ*** but not the in-plane angle ***ϕ***, which is calculated from individual experimental intensities at different polarisations according to ref. 26. Such corrections are not needed in the case of ***ϕ***, because only the ratio of differences of intensities appears in equations for ***ϕ*** and all offsets and normalisations therefore cancel out.

As discussed in the previous section, the sign of *n*_*z*_ cannot be determined from the experiment. This means that in a naive reconstruction there are domains of director with wrong vertical orientation as can be seen in areas marked with an orange ellipse in Fig. 1f. Before running the simulated annealing algorithm to determine the correct signs of *z* components, regions outside the droplet were filled with a director field with a radial configuration pointing toward the centre of the droplet, therefore acting as effective homeotropic anchoring on the surface of the droplet. Finally, we applied the simulated annealing algorithm and reconstructed the director inside the droplet, which is shown in two cross sections in Fig. 1g,h. No *z*-director sign artefacts are seen in either of the projections and twisting around the central point defect is clearly seen in Fig. 1h, relaxing some of the splay deformation^38^.

An example of FCPM study of a complex droplet and the director field, reconstructed from it, are presented in Fig. 1i–m. Figure 1i–l show experimental intensities for the four polarisations of excitation and detection with bleaching correction and deconvolution applied. The intensities correspond to spatial variation of the director field: in areas of high intensity the director is mostly in the *xy* plane and pointing approximately in the direction of the used polarisation. The reconstructed director structure, calculated from the experimental data in Fig. 1i–l and annealed with the presented algorithm, is shown in Fig. 1m. Director structure of this type is analysed in detail in the following section (the case with *N* ≈ 3.0).

## Results and Discussion

The parameter which determines the topological complexity of structures in chiral nematic droplets, is the chirality parameter *N* = 2*d*/*p*_0_, where *d* is the diameter of the droplet and *p*_0_ is the intrinsic pitch of the chiral nematic LC. Pitch is the distance, over which the director rotates by 360°. In the following we present examples of numerically reconstructed 3D director profiles from 3D FCPM imaging of chiral nematic droplets with *N* increasing up to 6.

**Figure 2:**
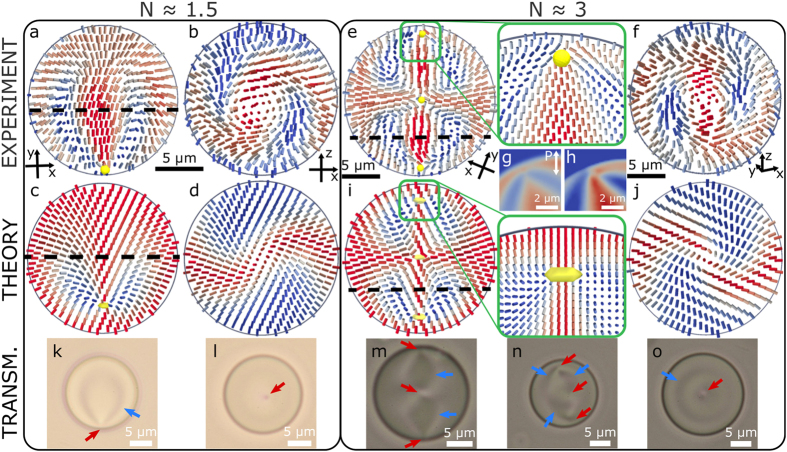
Comparison of experimentally reconstructed and numerically simulated director structures in chiral nematic droplets with one and three point defects. (**a**) Cross section of the reconstructed director field in a droplet with a single point defect in the equatorial plane at *N* ≈ 1.5. (**b**) Cross section taken along the dashed line in (**a**). (**c**) Numerical simulation for *N* ≈ 1.5 gives the same topology as experimentally observed in (**a**). (**d**) Cross section along the dashed line in (**c**). (**e**) Three collinear point defects are observed in the reconstructed director of a droplet with increased chirality parameter *N* ≈ 3.0. The inset shows a zoom-in of the off-centre point defect. (**f**) Reconstructed director in a cross section taken along the dashed line in (**e**). (**g**) The intensity of a single polarisation P close to the upper defect in (**e**). (**h**) Total FCPM intensity of the defect in (**g**). (**i**) Numerically calculated director corresponding to the reconstructed director in (**e**) at *N* = 2.0. (**j**) Cross section taken along the dashed line in (**i**). All cylinders are coloured by *I*_norm_ or the size of the director projection on the *xy* plane. In experimental images the point defects are marked with yellow spheres and in theoretical ones with yellow isosurfaces corresponding to *S* = 0.5. (**k**–**o**) Transmission microscopy pictures of droplets with different orientations of the symmetry axis. Panels (**k**,**m**) show droplets with the symmetry axis in the image plane, (**l**,**o**) perpendicular to the image plane and, (**n**) with the symmetry axis tilted out of the image plane. Locations of point defects are marked with red and of cholesteric layers with blue arrows.

In the case of a low-chirality droplet (*N* ≈ 1.5), the 3D reconstructed director profile shows that the point defect is expelled from the centre of the droplet, where it was residing for zero chirality, and the central region is filled with a twisting structure, as shown in Fig. 2a,b. By examining the reconstructed director field in a plane perpendicular to the symmetry axis of the droplet (Fig. 2b) we can see that the director twists along any diametrical line by almost 2*π* with some additional bending to satisfy the boundary conditions. This indicates that the local structure is a double-twist cylinder and in the *xz* cross section resembles a two-dimensional skyrmion^27^, known as the Bloch-type skyrmion in chiral magnets^39^. It is bound by a point defect on one end and connects to the droplet surface without discontinuities in the director field on the other. Figure 2c,d present corresponding numerical simulation of the director with *N* ≈ 1.5, which shows good agreement with reconstructed experimental images. Non-polarized transmission images of droplets with the same type of structure as in Fig. 2a,b are shown in Fig. 2k,l. One can clearly observe a single point defect per droplet, which appears as a dark spot, as well as twisted cholesteric layers which act as optical lenses and therefore appear as dark elongated patches.

In a droplet with higher chirality parameter (*N* ≈ 3.0), shown in Fig. 2e,f, we observe three collinear point defects, connected with two skyrmion-like profiles. This skyrmion profile can be seen from the cross section, taken along the dashed line in Fig. 2e and shown in Fig. 2f. The two skyrmions share a hyperbolic point defect, located in the centre of the droplet. Because the droplet surface is curved, the director on the skyrmion-like 2D cross sections (dashed lines in Fig. 2a,c,e,i) spans a little less than all possible spatial directions, but the boundary condition on the droplet surface ensures its topological stability in the same way a uniform far-field would, as the only way of removing the skyrmion is to move a point defect across the cross-section.

The near-surface point defects in both droplets are different from simple radial or hyperbolic hedgehogs, as can be seen from the reduction of total fluorescence *I*_norm_ around the defects, shown in the insets to Fig. 2e,i. This intensity variation clearly signifies the twisting of the director out of the *xy* plane. The inset to Fig. 2e reveals that the reconstructed director profile around such a defect is similar to a shrunken twist disclination obtained with numerical relaxation (inset to Fig. 2i). By further examining the single polarisation experimental image (Fig. 2g) we conclude that the twisting around the defect is confined to a small region and the defect is a twisted point defect, well separated from the surface. The sum of experimental intensities *I*_norm_, shown in Fig. 2h, is considerably more smeared out around the defect than the single polarisation image, which indicates a short-coming of the FCPM method in reconstructing director fields in NLC – sharp features can be obscured by the inherent fluctuations of the LC and movements of the structures between polarization scans. Because of this it is often better to determine exact locations of defects from experimental FCPM intensities and not from the reconstructed director field which may contain artefacts.

By comparing the experimentally reconstructed director fields (upper set of panels in Fig. 2) with the corresponding numerical simulations (middle row of panels in Fig. 2) we can see a striking agreement, as the number and positions of point defects as well as equivalent topology are reproduced. There are minor differences due to more twisted experimental structures, which are due to the difference in sizes of real and much smaller simulated droplets (~15 μm vs. 2.5 μm), and the lower twist constant of the real LC material. Despite these disagreements, our new experimental procedure clearly gives topologically consistent results and can serve as a stand-alone method to reconstruct director structures from raw FCPM data.

**Figure 3:**
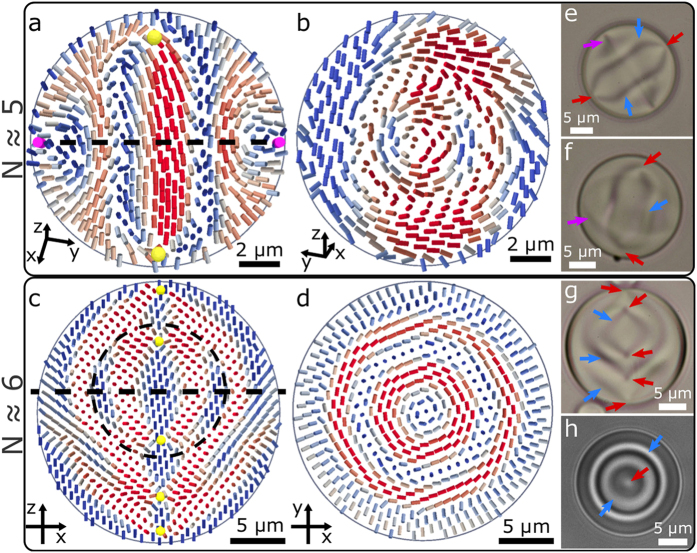
Points and loops in droplets with higher chirality. (**a**,**b**) The director field, reconstructed from 3D FCPM images in two perpendicular cross sections in a chiral nematic droplet with *N* ≈ 5. Two point defects (yellow dots) and a disclination ring (purple dots) can be seen in (**a**). The cross section shown in (**b**) includes the disclination ring. (**c**) A different droplet with *N* ≈ 6, showing a total of 5 point defects (yellow dots) with the region inside the dashed circle corresponding to a toron. In this cross section, the elongation of the droplet along the *z* direction is an optical artefact due to refractive index mismatch between the LC and surrounding medium, as well as the birefringence of the LC. In this case the effect is pronounced because of the size of the droplet and the type and particular orientation of the director structure. (**d**) The reconstructed director field in a cross section located at the dashed line in (**c**). The colours of cylinders indicate *I*_norm_ or the size of the director projection on the *xy* plane. (**e**–**h**) Transmission microscopy pictures of droplets with different orientations of the symmetry axis. Panels (**e**,**g**) show droplets with the symmetry axis in the image plane, (**f**) with the symmetry axis at an oblique angle and (**h**) perpendicular to the image plane. Locations of point defects are marked with red, disclination lines with purple, and of cholesteric layers with blue arrows.

In droplets with even higher chirality (*N* ≈ 4–6), similar skyrmion-like structures are found, but with additional layers of twist. In Fig. 3a, which shows the reconstructed director field in a droplet with two point defects and a disclination loop, the central part between the two point defects is a typical double-twist cylinder. In the plane of the disclination loop (Fig. 3b) the director twists by 3*π* across the diameter, as opposed to 2*π* in droplets with lower chirality. Because of this additional twist the director does not match the anchoring conditions on the droplet surface and a twist disclination ring is needed to stabilise the structure. In a droplet with a slightly higher chirality (Fig. 3c,d) we observe five point defects and even more twist (4*π*) occurs in the two-layered skyrmionic structure which includes the top four point defects. Because the amount of twist fits the anchoring around the structure, no disclinations are needed. The structure is similar to diametric spherical structure in droplets with planar anchoring^16^, but due to the different anchoring condition, another smaller skyrmionic layer with an associated point defect completes the structure. The inner-most layer, marked with a dashed circle in Fig. 3c, structurally resembles a toron and its less twisted relatives^25^,^27^, but is nested within outer concentric layers instead of uniform director far-field, observed in unwound cholesteric liquid crystal in homeotropic cells.

Nematic point defects are classified with an integer invariant called the topological charge, which measures how many times the full set of possible spatial orientations of the director occurs in the vicinity of the defect, provided we represent the director as a vector field^40^. The topology of a sphere with homeotropic anchoring forces the defects inside to have a total of +1 topological charge, which is realized as a single defect in the case of an achiral nematic droplet^9^,^41^. In our droplets, multiple defects are stabilised by twisted cholesteric areas, which provide elastic repulsion between them. If we make the director a valid vector field by decorating it with arrows continuously, so that they point outwards on the droplet surface, we observe that adjacent point defects in presented structures with 3 and 5 point defects have opposite topological charges. As long as no entangled disclination loops or higher order defects are present, an odd number of point defects will always be observed, consisting of alternating charges interspaced with skyrmionic layers, totalling to a charge of +1. The elastically twisted medium deforms the point defects from simple radial or hyperbolic hedgehogs into intermediate twisted textures. Because of this it is not sufficient to infer the signs of topological charges from the geometry of the director around the defect, but instead the director field as a whole must be considered to assign the topological charge signs consistently, as illustrated in refs 26 and 40.

In chiral nematic droplets presented here, we have observed local structures, which resemble skyrmions and torons. However, it is misleading to classify these as isolated objects. In extended homeotropic cells, torons, hopfions, skyrmions and finger loops behave as independent freely mobile quasi-particles in homogeneous director far-field and can thus be treated as separate entities. Similarly, in bulk cholesteric samples, it is easy to define the direction of a helical axis and point out the positions of well known objects, such as double-twist cylinders and cholesteric disclinations. On the contrary, the strongly confined and closed environment of a droplet makes such classification difficult. Not only are the “torons” and “skyrmions” tightly merged into a single continuous texture, but the presence of point defects and achiral boundaries makes the local helical axis ambiguous and in some regions undefined. Following the definition in ref. 42 the pitch axis is the direction to which the director is locally perpendicular and rotates around it. If the director does not locally resemble the cholesteric ground state, no such direction can be found. Just like the director field is undefined at nematic defects and at regions of isotropic phase, the inability to define the pitch axis on a thin tube-like region signifies a cholesteric defect line and extensive 3D regions without a pitch axis must be regarded as regular achiral nematic. It should be noted that for general director fields, several different definitions of the pitch axis can be formulated, with slightly different results in the regions significantly dissimilar from the cholesteric ground state^42^,^43^.

**Figure 4:**
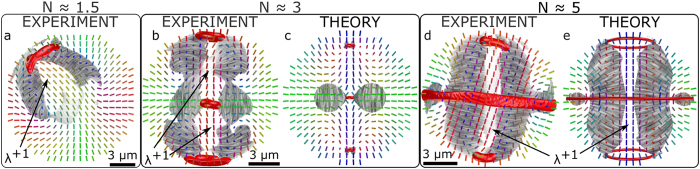
Comparison of experimental and numerically obtained structures by analysis of the pitch axis. Gray streaks show the pitch axis orientation in the plane of the image. Red areas contain singular point and line defects. (**a**) A low chirality droplet from Fig. 2a,b, where the reconstructed director was slightly smoothed to increase the numerical stability of the calculation of spatial derivatives. The distorted part near the defect qualitatively resembles a double-twist cylinder with its radially oriented pitch axis, but the rest of the droplet has mostly splay deformation and no helical order. The corresponding theoretical simulation, depicted in 2c,d is not shown, as it contains no purely cholesteric regions due to the simplified elastic constants in simulation. (**b**) A structure from Fig. 2e,f shows the pitch axis of two skyrmion-like structures, but the middle part twists along the symmetry axis, due to the twist-like nature of the central point defect. A large outer region is still too deformed to be described as cholesteric. The corresponding numerically obtained structure in (**c**) exhibits much less twisting in the radial direction, but still has cholesteric regions around the central point defect. (**d**) In the droplet from Fig. 3a,b, a larger part of the droplet exhibits clear helical order with cylindrical symmetry and shows good agreement with the numerical structure (**e**). The pitch axis is undefined along the symmetry axis in all the presented structures, which can be interpreted as the core of a *λ*^+1^ disclination line – i. e. the double-twist cylinder. Note that the position of the boundaries where the pitch axis becomes undefined is very sensitive to distortions produced during FCPM measurement and later stages of the reconstruction.

To illustrate the identification of cholesteric features, we calculated the local pitch axis in experimental and numerical structures following the definition from ref. 42 (Fig. 4). Because the calculation involves spatial derivatives of the director field, we had to smoothen the experimentally reconstructed director field by running it through a few steps of the same numerical relaxation, as used for the numerical simulations. This elastically relaxed the reconstructed structures, smoothed sharp jumps and artefacts, and increased the stability of numerical derivatives in the calculation of the pitch axis. Because the implemented numerical relaxation is tensorial and therefore includes the scalar order parameter, the noisy director field around defects melts to isotropic phase. As a result, point defects appear as somewhat larger isotropic regions after this procedure. Figure 4a,b,d show the extent of cholesteric regions computed from the experimentally reconstructed director field, with smoothing applied to allow numerical calculation of derivatives. In addition to the central *λ*^+1^-like non-singular disclinations along the symmetry axes of droplets which connect the point defects, large non-cholesteric regions are found near the homeotropic boundaries, especially in droplets with lower chirality. It is instructive to compare the pitch axis found on experimental data with the numerical ones. For the lower two chiralities the numerically obtained structures (Fig. 4c) are much less twisted than the experimental ones and large parts of the droplet are dominated by bend instead of twist. As explained before, this is a consequence of the differences in size of the droplets and elastic constants in the experiment and the numerical simulation, both of which can change the relative energetic cost of the different elastic deformations just enough that the pitch axis is undefined by the definition we utilized. What is intriguing is that even with less twist deformation the director field in the cross-section of numerical droplets is still skyrmion-like, but resembling the Néel-type skyrmion^39^ instead of the Bloch-type as in the experiment. Chirality in this system is therefore not as important to energetically stabilise the skyrmionic structure as it is to break the symmetry which induces the formation of either of the two types of skyrmions.

The reconstructed director fields from our 3D FCPM images give an important insight into the visual appearance of simple optical transmission images, which were recently used to illustrate the richness of topological structures in chiral nematic droplets^21^. Because the director structures in droplets can be oriented in any direction, the droplets can exhibit a continuous variety of textures. Some examples of textures for different orientations of the presented structures are shown in Figs 2k–o and 3e–h. Locations of defects and cholesteric layers can be seen in these micrografs, but caution is needed when interpreting the images because they are 2D projections of 3D director fields. This causes problems, when the director field is strongly changing along the optical axis, e. g. when the symmetry axis is parallel to the optical axis as in Figs 2m and 3h where several point defects are obscured. Another point of caution is the difficulty of discerning cholesteric layers from line defects (e. g. Fig. 3e,f), especially in liquid crystals with higher birefringence, where the lensing effects are enhanced.

## Conclusions

We successfully determined for the first time the full three-dimensional director field from raw experimental FCPM data on chiral nematic droplets using new FCPM director reconstruction method based on simulated annealing of director field. Unlike most cholesteric structures with simple twisting along a single helical axis, the cholesteric droplets contain fundamentally three-dimensional director field textures, which were robustly reconstructed with our technique. We also demonstrated that in moderately chiral systems with appropriate dimensions, the optical resolution of the FCPM is not the limiting factor for detailed reconstruction of structures, because thermal fluctuations of defects and movements of the sample cause larger de-focusing and error. Although the described algorithm was used on data obtained with FCPM it could be easily applied to any other polarisation sensitive microscopy method for obtaining the orientational order of matter.

This method opens a new avenue of future studies of topologically complex structures of liquid crystals and allows for testing of universal concepts of topology of fields in frustrated environments. A point of special interest is the characterization of the twisted skyrmion-like structures in the droplets. In the frustrated environment, these structures are testing the boundaries of our understanding of cholesteric textures and require careful theoretical investigation. Although the current study is focused on droplets of moderate chirality of around 2–6 turns per diameter, we anticipate that more exotic structures will be readily discovered and classified in future experiments. Interestingly enough, we have observed no linked and knotted defect loops, as predicted by theory^17^. The reason for this is in the high energy cost, which is required for the formation of molten defect cores for this size of droplets. This energy barrier could be lowered by performing the experiments close to the isotropic phase transition, by increasing the chirality and by using temperature quench to reach metastable topological states.

## Methods

### Sample preparation

The LC mixture was prepared by mixing equal amounts of calamitic liquid crystals CCN-47 and CCN-55 (refractive indices of the mixture *n*_*o*_ = 1.47, *n*_*e*_ = 1.50). This mixture exhibits nematic phase at room temperature but phase separates at temperatures below 15 °C and goes into isotropic phase at around 60 °C. Its low birefringence (Δ*n* = 0.03) minimises artefacts caused by defocusing and polarisation guiding of the excitation and fluorescence light^24^. The mixture was doped (typically 1–2% wt.) with chiral dopants S-811 for left-handed and with CB15 for right-handed mixtures of suitable pitch (typically 5–10 μm). For FCPM microscopy this mixture was additionally doped with small amounts of fluorescent dye BTBP which was first disolved in acetone, then added to the LC mixture to achieve a homogeneous isotropic mixture and left at room temperature to evaporate the acetone. Droplets were prepared by mixing small amounts (typically a few percent) of the LC mixture into glycerol with 4 wt.% of L-*α*-phosphatidylcholine acting as a surfactant to ensure homeotropic anchoring on the LC/glycerol interfaces. Glycerol was chosen as a carrying medium because of low refractive index mismatch compared to the LC mixture (*n* for glycerol is 1.474) minimising the spherical aberrations and because of its high viscosity. The LC/glycerol mixture was sandwiched between a 150 μm cover glass and a 1 mm glass plate separated by a 30 μm spacer and sealed with a two-component epoxy glue to minimise the movements of the droplets due to flow.

### FCPM measurements

For the FCPM experiments a Leica TCS SP5 X confocal system based on an Leica DMI6000B inverted motorised microscope was used. A narrow band (apprx. 1–2 nm) of wavelengths was selected from Leica WLL continuous spectrum pulsed laser by an acousto-optic transmission filter and joined to the main beam path using an acousto-optic beam splitter (AOBS). The beam was deflected by two galvo mirrors moving in perpendicular directions to perform the XY scanning. A quarter wave-plate was used to transform the polarisation of the excitation light from linear to circular. By adding a subsequent polariser it was possible to select any linear polarisation. The excitation beam was focused on the sample through a high numerical aperture oil immersion objective (Leica HCX PL APO lambda blue 63.0 × 1.40 OIL UV). The fluorescence light was collected through the same objective and passed the same polariser, wave-plate and galvo mirrors but continued straight through the AOBS after which it was dispersed by a prism and a band of wavelengths was selected by an adjustable slit to pass to a photomultiplier. Light with a wavelength of 488 nm was used to excite BTBP and fluorescence was detected in the band 515–575 nm. Fluorescence intensities were taken at four polarisations of excitation and detection (0, *π*/4, *π*/2, 3*π*/4) and then the scan at the 0 polarisation was repeated to correct for bleaching. Differences in intensities between the two scans at 0° were between 10 and 50% and the bleaching rates were linearly interpolated for all the polarisations so that the integral intensities of the droplet in the first and last scan were equal. After bleaching correction deconvolution was preformed on the data using SVI Huygens software and the intensities at the four polarisations were summed to obtain *I*_exp_.

### Numerical modelling

Numerical simulations were performed with a relaxation minimization of the Q-tensor Landau-de Gennes free energy model with a single elastic constant and chirality contribution,


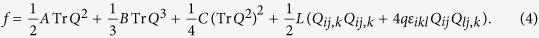


The elastic contribution matches the model used in post-processing of experimental data with simulated annealing. The well-tested material constants introduced in ref. 44 were used, as the goal of the simulation was to qualitatively confirm the experimental director field reconstruction. The inverse cholesteric pitch *q* was varied to produce droplets with different numbers of helical windings per diameter. A lattice size of 300 × 300 × 300 at 8 nm resolution was used.

## Additional Information

**How to cite this article**: Posnjak, G. *et al*. Points, skyrmions and torons in chiral nematic droplets. *Sci. Rep.* **6**, 26361; doi: 10.1038/srep26361 (2016).
